# Combining the optimization of the CT examination process with a vial sharing strategy for contrast agents: an economical and practical method for hospitals

**DOI:** 10.1186/s12913-026-15251-1

**Published:** 2026-07-30

**Authors:** Yanchao Yin, Wei Fu, Lin Qiu, Feie Li, Dong Liu, Juan Li

**Affiliations:** 1 Department of Pharmacy, Tongji Hospital, Tongji Medical College, Huazhong University of Science and Technology, Wuhan, 430022 China; 2 Pharmaceutical Department, Hubei Cancer Hospital,Tongji Medical College, Huazhong University of Science and Technology, Wuhan, 430079 China

**Keywords:** Drug waste, Vial-sharing, Contrast agent, Computed tomography (CT)

## Abstract

**Background:**

Drug wastage is a global concern that not only imposes an economic burden on healthcare systems but may also lead to inefficient resource allocation and environmental degradation, specifically through pharmaceutical residues in wastewater and carbon emissions from incineration. To address this issue, this study optimized the computed tomography (CT) examination process through multidisciplinary collaboration and changed the billing method for contrast agents from single-vial billing to vial-sharing billing. We aimed to quantify the difference in drug cost and waste volume between single-vial billing and vial-sharing billing for CT contrast agents in a tertiary hospital.

**Methods:**

This retrospective study was conducted at Tongji Hospital of Huazhong University of Science and Technology, Hubei province, China. Data were obtained from the Hospital Information System (HIS) for contrast-agent prescriptions from July 2023 to June 2024. We collected information on 9 types of drugs, including their names, unit prices per vial, drug specifications, and actual dosages. Drug costs and waste volumes were calculated using hospital procurement price lists.

**Results:**

The multidisciplinary optimization of the CT examination process was implemented. Via the vial-sharing billing method, a total of 2,906,398.8 mL of contrast agent was saved annually, corresponding to a cost reduction of 9,181,958.83 CNY. On average, each patient’s prescription cost was reduced by 66.93 CNY.

**Conclusion:**

The vial-sharing strategy has substantially reduced drug waste, alleviated the economic burden on patients, improved drug utilization efficiency, and conserved medical resources.

## Background

In recent years, drug shortages have not only constituted a prevalent global problem, but have also evolved into a complex global public health challenge [1–3]. Nevertheless, when drugs are in short supply, the phenomenon of drug waste remains widespread [2, 3]. The problem of drug waste has emerged as a major global concern, imposing an economic burden on healthcare systems and potentially resulting in inefficient resource allocation and environmental hazards [4]. In 2018, it was reported that the U.S. Centers for Medicare & Medicaid Services expended $725 million on medications that were discarded after being administered in hospital outpatient clinics and physician offices [5]. In England, the annual estimated cost of wasted medications in primary and community care settings is around £300 million, with nearly one-fifth of these wasted medications remaining entirely unused [6, 7].

Studies have indicated that drug waste arises from multiple factors, including excessive prescription, inappropriate storage management, expired medications, and patients’ non-adherence to medical recommendations [8–11]. Consequently, numerous countries have implemented a series of measures to reduce drug waste. For instance, in the United Kingdom, the National Health Service (NHS) implemented various strategies, including restricting prescription lengths, conducting medication reviews, encouraging the use of patients’ own medications, and introducing repeat dispensing schemes [6, 7]. Similarly, in the United States, the Food and Drug Administration (FDA) recommends that sponsors of pivotal trials for new or extended therapeutic indications use fixed dosing for a specific clinical indication [12]. Also, in China, the National Health Commission and the State Administration for Market Regulation jointly issued the “Implementation measures for saving drug resources and containing drug waste” in 2023 [13]. While these national guidelines provide a critical regulatory framework, their implementation in clinical settings remains inconsistent. Consequently, the problem of medication wastage persists in many hospitals, as systemic barriers often hinder the transition from policy to practice. Therefore, some medical institutions have implemented vial-sharing strategies, especially in the management of high-cost medications [14, 15]. This strategy has demonstrated remarkable effectiveness, capable of reducing drug waste and associated costs.

Vial-sharing has shown the largest and most consistent reductions in high-cost drug waste. Its core mechanism of vial sharing involves using residual medication from an opened vial for multiple eligible patients within the permitted in-use period to administer precise dosages, thereby minimizing waste and reducing costs. Multiple empirical studies [16–18] across different regions and drug types (patisiran, antimicrobials) have validated its efficacy: it achieves substantial cost savings (ranging from tens of thousands to millions in local currency) and significant reductions in drug discard volume or consumption. Additionally, vial sharing can alleviate the financial burden on patients’ families, potentially reducing financial barriers to diagnostic services. Overall, vial sharing holds great promise for optimizing healthcare resource utilization, easing the financial pressure on healthcare systems, and enhancing the accessibility of treatments.

Although the success of vial-sharing for high-cost medications in oncology has been well-documented, the application of vial-sharing strategies to contrast agents in medical imaging remains an underexplored area. In the context of the Chinese healthcare system, while the Basic Medical Insurance (BMI) provides broad coverage, diagnostic examinations involving high-cost contrast agents often require varying levels of co-payment or out-of-pocket expenditure. However, the specific dosing requirements of contrast agents—often calculated by patient weight—frequently result in significant leftover volumes when single-dose vials are used, paralleling the challenges observed in other medical fields [19–21]. To bridge this gap, initial research focused on optimizing physical packaging. For instance, Robinson et al. [22] demonstrated that transitioning from single-use vials (100 mL or 150 mL) to large-volume multi-use packaging (500 mL) not only yields significant cost savings but also enhances the quality of contrast opacification. Similarly, a study by GE Healthcare (a medical diagnostic provider) [23] quantified these benefits, reporting a cost reduction of £1.21 (US$1.58) per patient when utilizing 500-mL packages instead of 100-mL ones. However, as large-scale packaging is not yet universally available or compatible with all institutional infrastructures, vial-sharing strategies offer a more flexible and immediate administrative solution for contrast agents.

Resolving the causes of contrast agent waste requires not only procedural workflow optimization but also a fundamental evaluation of hospital billing structures. This study optimized the computed tomography (CT) examination workflow via multidisciplinary collaboration and transformed the billing method of contrast agents from single-vial billing to vial-sharing billing. This work aimed to comparatively assess the two billing methods with respect to medication expenditure and contrast agent wastage. In addition, it comprehensively analyzed the respective advantages and limitations of each model. The outcomes of this study are expected to provide evidence for promoting the cost-effective use of contrast agent in clinical practice.

## Methods

### Study design

This study was a single-center, retrospective descriptive analysis at Tongji Hospital, Huazhong University of Science and Technology, Hubei, China. The prescription data were collected from the Hospital Information System between July 2023 and June 2024. This study did not compare two independent patient cohorts. Instead, the study descriptively compared the actual vial-sharing billing model with a theoretical single-vial billing model using the same eligible contrast-agent prescriptions. Under the theoretical single-vial billing model, each patient would have been charged for an entire vial according to the vial specification, regardless of the residual volume. Under the vial-sharing billing model, patients were charged according to the actual volume administered. The main outcomes were total contrast-agent volume saved, medication cost savings, and the proportions of saved volume and saved costs. No inferential statistical analysis was performed.

Inclusion criteria: All patients who received contrast agents in the catalog were included in this study. Exclusion criteria: Patients who did not use contrast agents, or those receiving contrast agents not included in the catalog. Therefore, the sample size was determined based on the total number of contrast-enhanced CT examinations performed during the study period. This study followed the guidelines outlined in the Declaration of Helsinki and was approved by Tongji Hospital’s Hospital Ethics Committee. An exemption for informed consent was approved by the Ethics Committee because only clinical data were collected.

### Selection of drug catalog

We conducted a one-hour focus group (*n* = 6 senior pharmacists and radiologists) to determine the optimal agent types and volumes by the quantitative analysis of historical CT data. As well, we conducted an on-site investigation to solicit opinions from the medical staff about safety and stability, workflow efficiency, and risk management. Based on the actual situation of the hospital, we selected 9 types of contrast agents and developed a catalog of drugs (Table 1).

**Table 1:** Catalog of drugs priced using the smallest unit

Drug name	Manufacturer	Drug specifications	Price (CNY)	Agreed pricing unit	Agreed pricing (CNY)
Iopromide Injection	Bayer Vital GmbH	37 g/100mL	330.95	5 mL	16.55
Ioversol Injection	Liebel-Flarsheim Canada Inc.	35 g/100mL	311.23	5 mL	15.56
Iodixanol Injection (I)	GE Healthcare AS	32 g/100 mL	349.60	5 mL	17.48
Iopamidol Injection (I)	Nanjing Chia-Tai Tianqing Pharmaceutical Company	37 g/100mL	178.00	5 mL	8.90
Iopamidol Injection (II)	Beijing Beilu Pharmaceutical Co., Ltd.	37 g/100mL	91.30	5 mL	4.57
Iohexol Injection	Yangtze River Pharmaceutical Group	30 g/100mL	106.50	5 mL	5.33
Iomeprol Injection	Shanghai Bolaike Xinyi Pharmaceutical Co., Ltd.	40 g/100mL	463.80	5 mL	23.19
Iodixanol Injection (II)	Jiangsu Hengrui Pharmaceuticals Co., Ltd.	32 g/100 mL	174.70	5 mL	8.74
Iodixanol Injection (III)	Zhejiang Starry Pharmaceutical Co., Ltd.	32 g/100 mL	185.01	5 mL	9.25

Our institution is a tertiary hospital in China. The financing of medical services relies on the Basic Medical Insurance (BMI) scheme, which provides standardized reimbursement for contrast agents, and patient co-payments for out-of-pocket expenses. Under national regulations, hospitals must maintain transparent billing practices, which traditionally mandated that a single-use vial be charged to a single patient ID, regardless of the residual volume discarded.

We also clarified the agreed pricing unit for split billing drugs. For drugs whose prescribed quantities are inconsistent with specifications of the basic drug supply catalog, the agreed pricing unit was the minimum unit, or charges were based on the actual usage quantity. The prescription quantity, drug usage, and corresponding costs of these selected drugs were obtained through the Hospital Information System (HIS) for further analysis of costs and drug waste.

### Optimization of CT examination process and implementation of vial-sharing

#### Original CT examination process

**Fig. 1:**
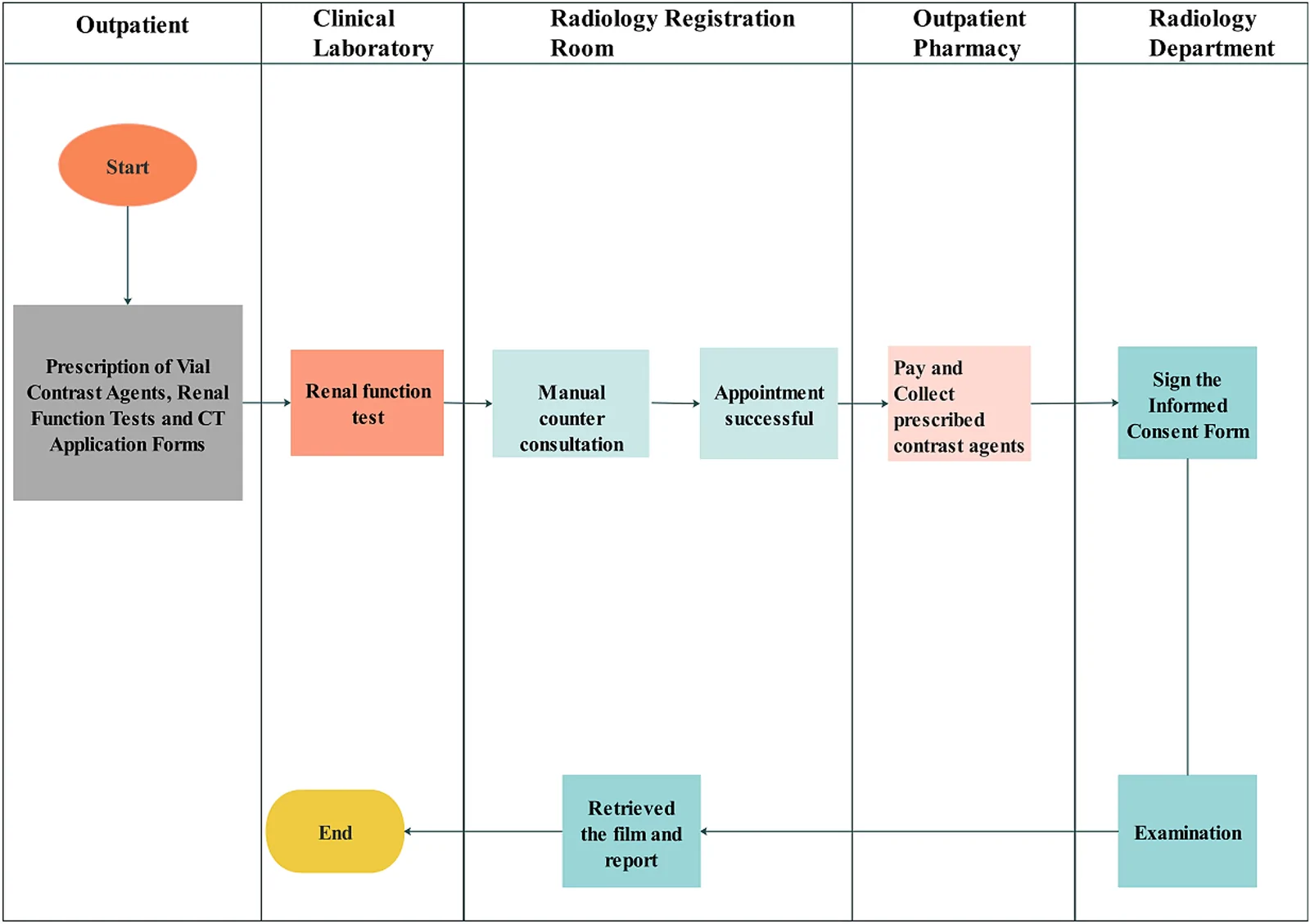
Original computed tomography (CT) examination workflow under the single-vial billing model. The workflow includes physician ordering, renal function testing, appointment scheduling, vial-based payment before examination, CT examination, 30-minute post-examination observation, and report retrieval

Figure 1 shows the original CT examination process. Firstly, the doctor issued a CT scan sheet, a renal function test in accordance with the European Society of Urogenital Radiology (ESUR) Guidelines on Contrast Agents (Version 10.0), and medication prescription. Then, the patient’s blood was drawn to check renal function. If the renal function results were normal, the examination was scheduled by staff in the radiology registration room. The examination fee and medication fee (vial) were then charged. Patients underwent examination at the scheduled time and location. After the examination was completed, the patients were observed for 30 min and left the examination room if there were no adverse reactions. Finally, the patient received the report.

#### Optimization of CT examination process

**Fig. 2:**
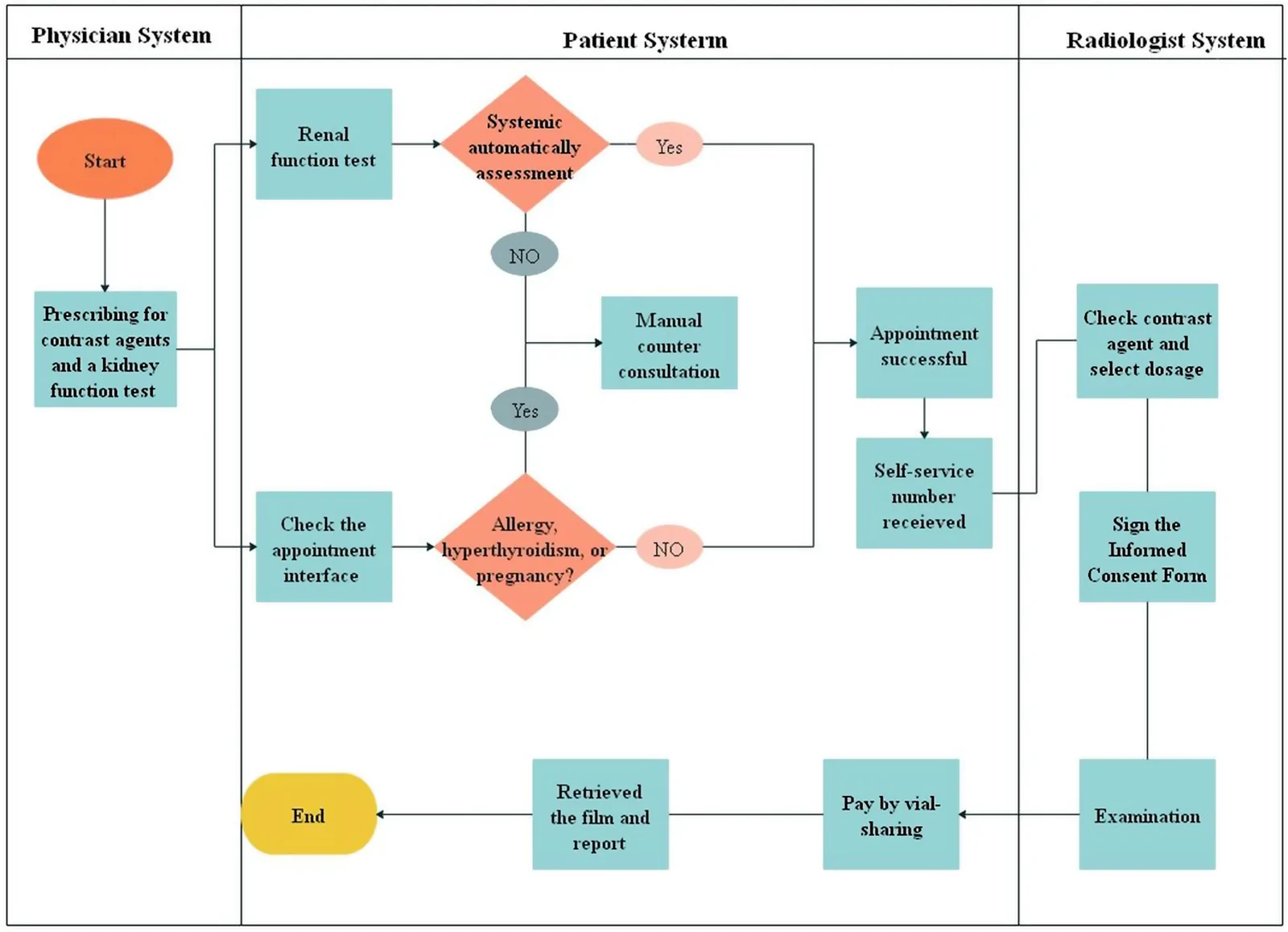
Optimized CT examination workflow under the vial-sharing billing model. The revised workflow integrates clinical assessment, renal function review, online appointment scheduling, individualized contrast-agent selection and dosing, post-examination observation, and actual-volume billing through multidisciplinary collaboration among clinical, laboratory, radiology, pharmacy, finance, and information technology departments

Through joint efforts by the finance, pharmacy, information technology, and radiology departments, we have transformed the process of drug billing from whole vial to vial-sharing. The flow chart is shown in Fig. 2. Except for the pharmacy department, multiple departments have played important roles. Specifically, the clinical departments (such as Oncology and Gastroenterology) initiated the process by evaluating clinical indications and documenting patient risk histories. Subsequently, the clinical laboratory department provided the essential biochemical data, specifically serum creatinine and eGFR, required for nephrotoxicity risk stratification. Finally, the department of radiology calculated the precise, individualized contrast volume required based on the specific imaging protocol and the patient’s body weight. The finance department deferred charging until completion of the CT examination. Also, the information department modified the appointment system.

Firstly, the doctor orders a CT examination and a renal function test, and does not need to select drugs or dosages on the system. The patient makes an online appointment after completing the renal function test. The automated scheduling interface incorporated a mandatory safety questionnaire addressing three foundational clinical variables. Specifically, the system required the confirmation of the patient’s allergy history, hyperthyroidism status, and pregnancy status. The system automatically retrieves patients’ renal function test results. Appointments are successfully confirmed if there are no contraindications and the test results are normal. If contraindications exist or abnormal renal function indicators are detected, patients need to consult staff at the service counter. During the examination, the patient collects their queue number at the self-service machine. A radiologic technologist selected the type and dose of contrast agent, and the patient signed the ‘Informed Consent Form’ before the examination. After the examination, the patient needs to be observed for 30 minutes. The 30-minute post-examination observation is a standard safety protocol at our institution to monitor for delayed adverse contrast reactions, in accordance with clinical safety guidelines. When the patient is under observation, they can pay the contrast agent fee (based on vial-sharing strategy) via a self-service kiosk or mobile phone. If there are no abnormalities during observation, they can leave the examination room.

#### Implementation of vial-sharing

To enhance patient safety and minimize contamination risk during the vial-sharing process, all contrast agent compounding and volume allocation procedures were performed within the contrast preparation room. The rubber stopper was disinfected with 75% alcohol before each puncture. When a vial was punctured, a sterile fluid-transfer spike was inserted into the rubber stopper. Individualized, patient-specific doses were then drawn into separate, single-use sterile syringes according to the precise volumes prescribed. A new single-use sterile syringe and connecting tube were used for each patient to reduce the risk of patient-to-patient cross-contamination. Furthermore, based on institutional aseptic-handling protocols and expert consensus recommendations, opened contrast vials were assigned a maximum in-use period of 4 h from initial puncture [24]. This 4-h limit was implemented as an aseptic-handling safeguard rather than being based on newly generated physicochemical stability or microbiological testing data from the present study. Any residual volume remaining in a vial exceeding this 4-h window, or at the end of a shift, was immediately discarded. However, this retrospective study did not include formal microbiological sterility testing, endotoxin testing, particulate testing, or physicochemical stability assessment.

### Charge mode

**Fig. 3:**
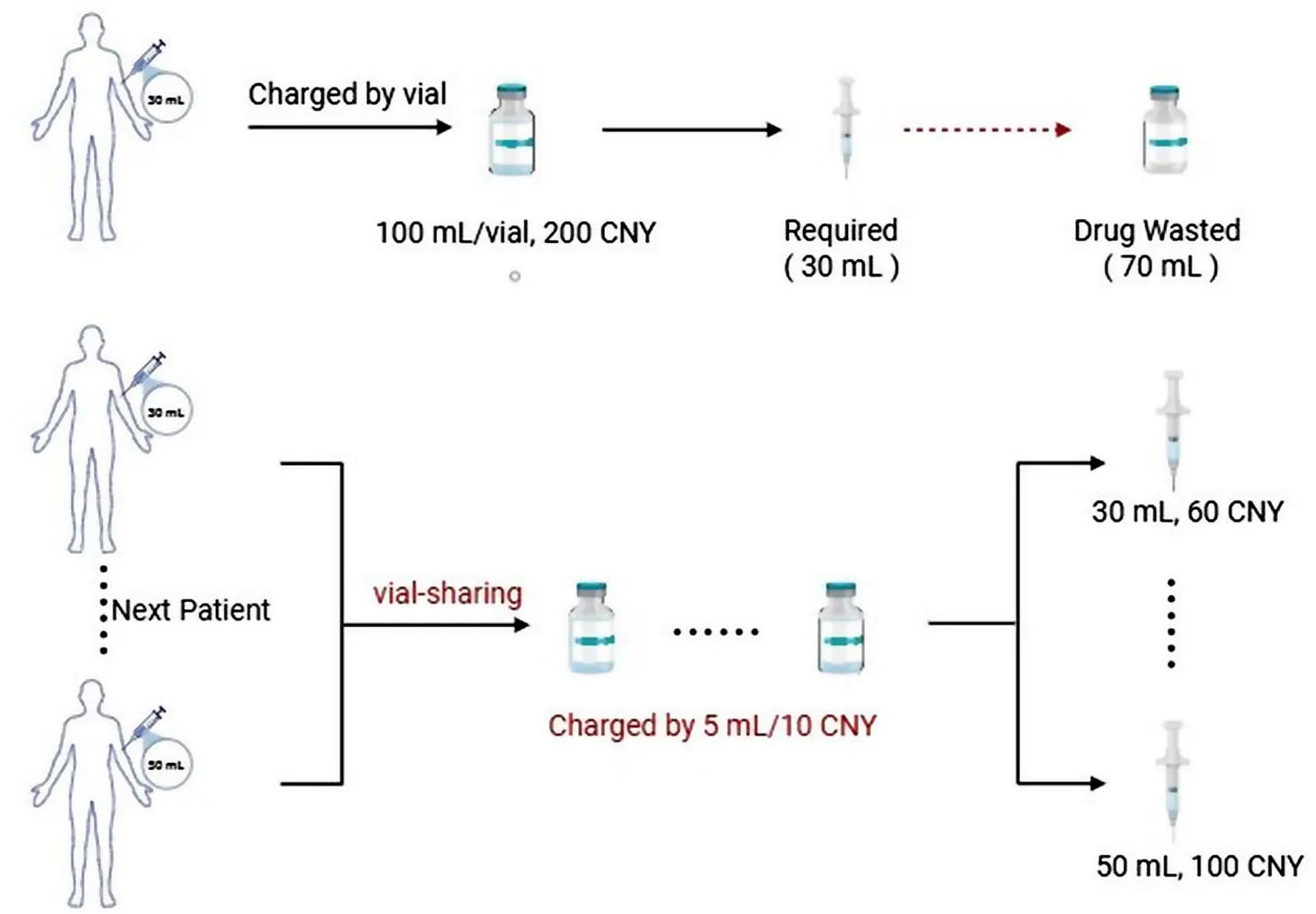
Infographic-style comparison of single-vial billing (Original mode) and vial-sharing billing (New mode). In the example shown, a patient requiring 30 mL from a 100-mL vial priced at CNY 200 would be charged for the whole vial under the original model, resulting in 70 mL of residual volume. Under the vial-sharing model, the patient is charged only for the actual administered volume according to the agreed minimum billing unit, reducing both patient expenditure and contrast-agent waste

Two CT examination processes correspond to two contrast agent charging methods. Under the original process, patients were charged by the vial. While under the optimized process, patients were charged according to the actual volume used and patients only need to pay for the dosage they used. For example, as shown in Fig. 3, the dosage of the patient is 30 mL, the specification of the drug is 100 mL, and the price is 200 CNY. The patient needs to pay 200 CNY for one vial, which also results in the waste of 70 mL of the drug. However, when implementing the vial-sharing strategy, each drug is split into the smallest billing unit according to the drug specifications, and patients are charged based on the actual usage unit. As shown in Fig. 3, the minimum pricing unit for this drug is 5 mL/10 CNY. This patient only needs to pay 60 CNY for 30 mL of the drug. The remaining medication can be shared with other patients thereby reducing waste.

### Data collection and calculation

Data were extracted from the HIS covering the period from July 2023 to June 2024. Information was gathered on nine types of drugs, including their names, unit prices, specifications, and actual dosages. Records with missing or inconsistent key variables, including contrast-agent name, specification, unit price, actual administered volume, or billing information, were checked against the HIS and excluded if the information could not be verified. The methodology employed in this study was adapted from established protocols for evaluating drug waste and cost-saving strategies in healthcare settings [18, 25]. The study calculated the total volume of drugs saved, overall waste costs, defined as the monetary value of the contrast agent volume that is lost through residual discard, and overall cost savings, defined as the cost savings between the theoretical cost under the traditional model and the actual expenditure under the vial-sharing model. The cost analysis was limited to direct medication expenditure calculated from prescription and billing data and the estimated contrast-agent volume saved. Implementation and operating costs, staff time, transfer devices, syringes, IT system changes, training, monitoring, and disposal costs were not included. Data analysis was performed using Microsoft Office Excel 2016.

## Results

### Number of medication prescriptions

**Fig. 4:**
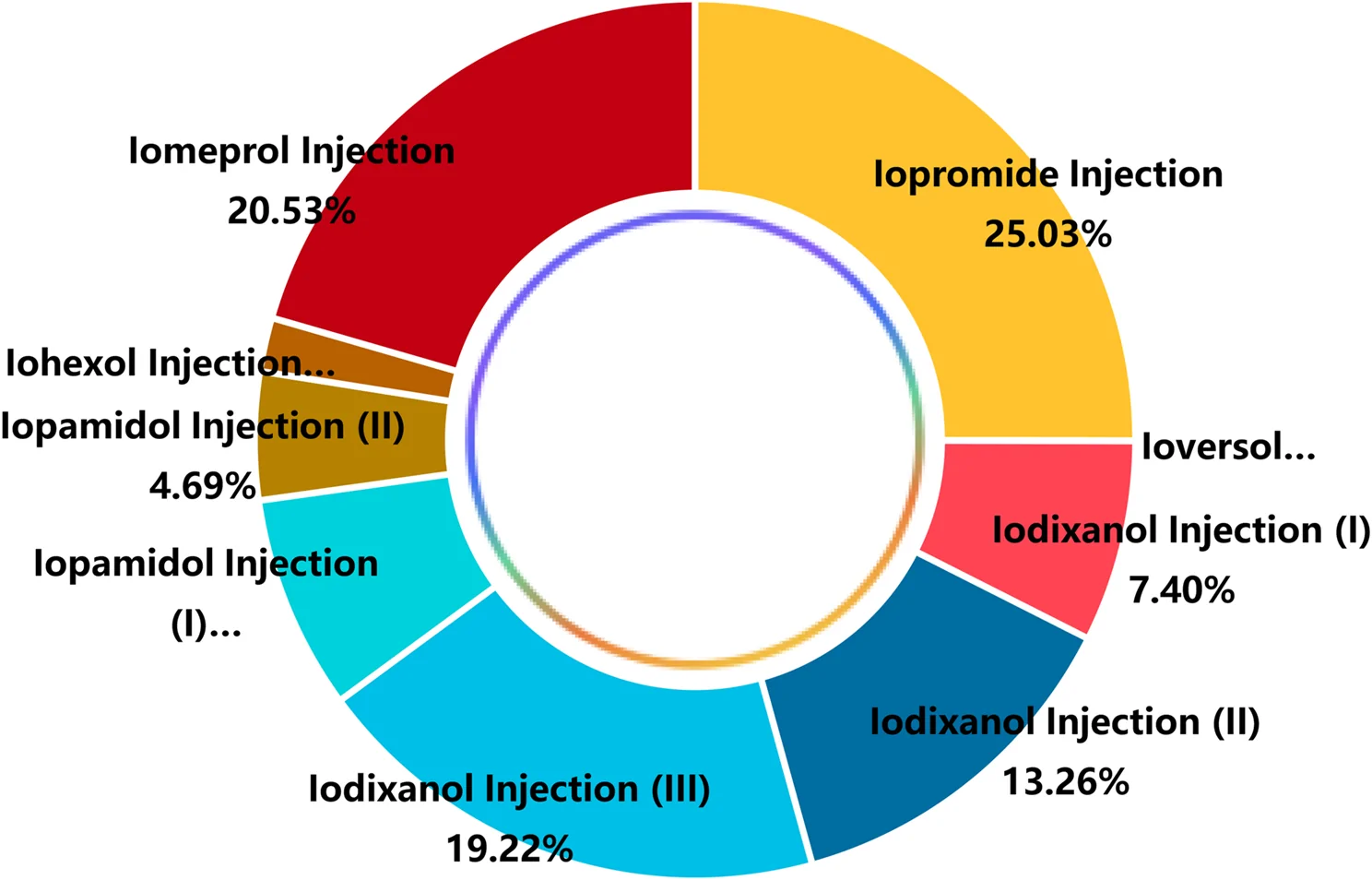
The percentage of drug prescriptions that use vial-sharing strategy

No missing data were identified for the key variables included in the final analysis, including contrast-agent name, specification, unit price, actual administered volume, and billing information. A total of 137,182 eligible contrast-agent prescriptions were included in the final analysis. Figure 4 illustrates the percentage of medication prescriptions that use vial-sharing strategy. The five leading drugs are as follows: Iopromide Injection accounts for 25.03%, Iomeprol Injection for 20.53%, Iodixanol Injection (III) for 19.22%, Iodixanol Injection (II) for 13.26%, and Iopamidol Injection (I) for 7.88%.

### Amount and cost savings

**Fig. 5:**
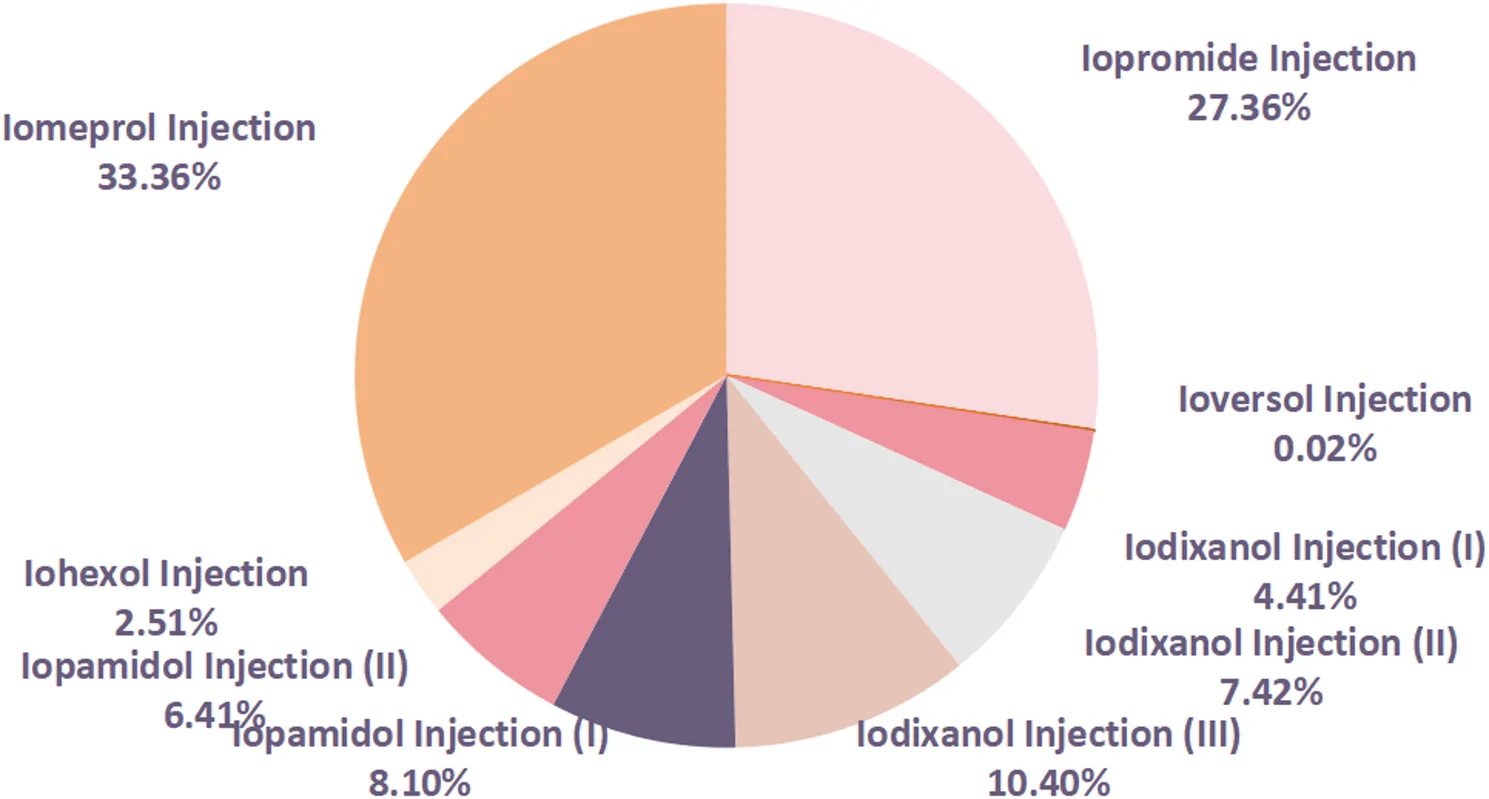
Proportion of total saved volume attributable to each contrast agent

The amount and direct medication cost savings results are shown in Table 2. The vial-sharing strategy effectively reduced contrast-agent volume and costs. Compared with the original single-vial billing model, the vial-sharing strategy saved a total of 2,906,398.8 mL of contrast agent. The proportion of saved volume is shown in Fig. 5. The top 3 largest saved volumes were Iomeprol Injection (33.36%), Iopromide Injection (27.36%), and Iodixanol Injection (III) (10.40%).

**Table 2:** Summary table of total prices

Drug name	Price (CNY)	Number of prescriptions	Original mode costs (CNY)	Actual Volume (mL)	Saved Volume (mL)	New mode costs (CNY)	Cost savings (CNY)	Proportion
Iopromide Injection	330.95	34,331	11,361,844.45	2,638,018	795,082	8,730,540.96	**2**,**631**,**303.49**	23.16%
Ioversol Injection	311.23	22	6,847.06	1600	600	4,979.62	**1**,**867.44**	27.27%
Iodixanol Injection (I)	349.60	10,157	3,550,887.2	887399.1	128300.9	3,101,839.36	**449**,**047.84**	12.65%
Iodixanol Injection (II)	174.70	18,190	3,177,793	1603325.5	215674.5	2,801,009.7	**376**,**783.3**	11.86%
Iodixanol Injection (III)	185.01	26,368	4,878,343.68	2,334,445	302,355	4,318,986.91	**559**,**356.77**	11.47%
Iopamidol Injection (I)	178.00	10,804	1,923,112	844,889	235,511	1,503,902.42	**419**,**209.58**	21.80%
Iopamidol Injection (II)	91.30	6,436	587,606.8	457,170	186,430	417,396.21	**170**,**210.59**	28.97%
Iohexol Injection	106.50	2,711	288,721.5	198,170	72,930	211,051.05	**77**,**670.45**	26.90%
Iomeprol Injection	463.80	28,163	13,061,999.4	1846784.6	969515.4	8,565,490.03	**4**,**496**,**509.37**	34.42%
Total		**137**,**182**	**38**,**837**,**155.09**	**10811801.2**	**2906398.8**	**29**,**655**,**196.26**	**9**,**181**,**958.83**	23.64%

**Fig. 6:**
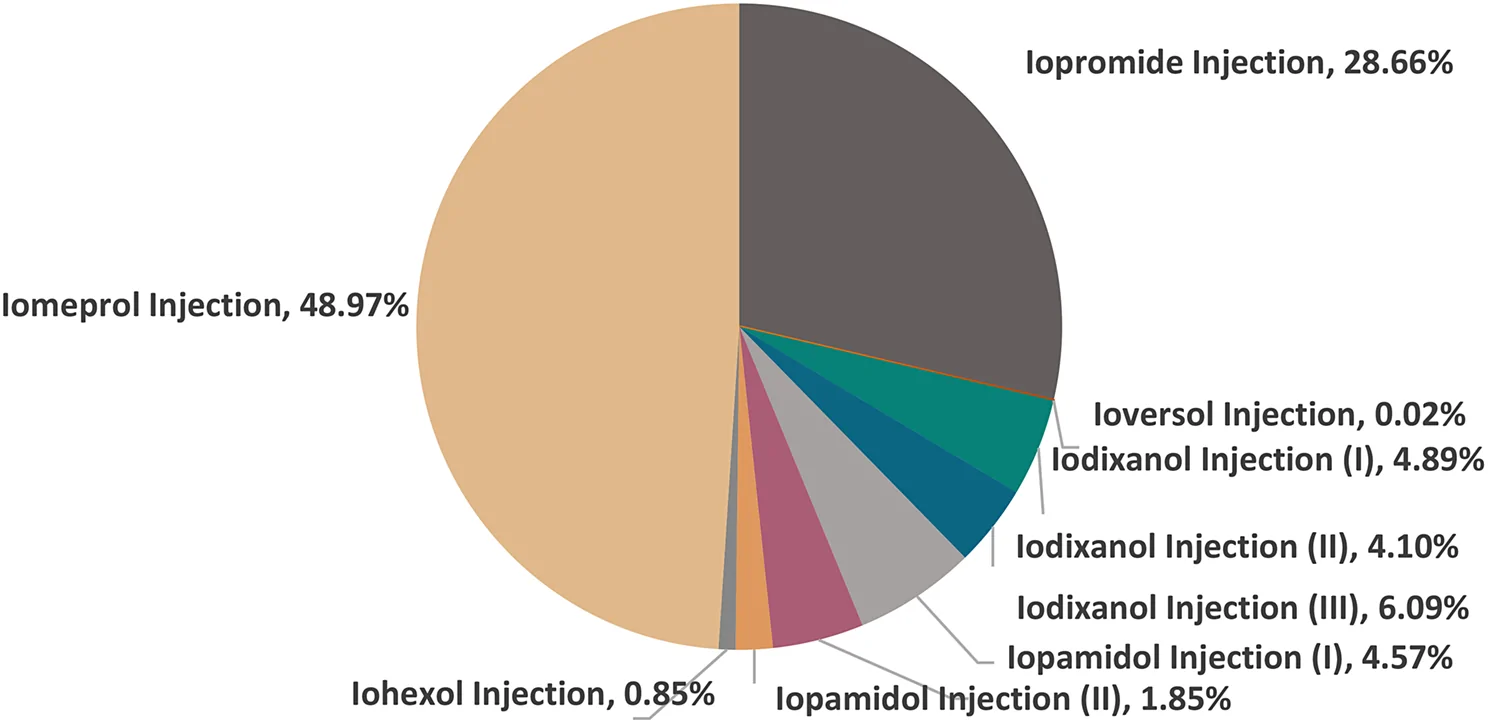
Proportion of total cost savings attributable to each contrast agent

Overall, total expenditure under the vial-sharing strategy was CNY 29,655,196.26, with direct medication cost savings of CNY 9,181,958.83 across the 9 contrast agents. The percentage saving was calculated as the cost saved divided by the theoretical cost under the original single-vial model. Through the vial-sharing strategy, 23.64% of drug costs were saved within one year. Also, the proportion of total cost savings attributable to each contrast agent was shown on Fig. 6. The top 3 cost-saved proportion drugs were Iomeprol Injection (48.97%), Iopromide Injection (28.66%), and Iodixanol Injection (III) (6.09%).

### Drug specification recommendation

We compiled the specifications of drugs billed under the vial-sharing strategy during the study period. And we aimed to identify the most frequently used drug specifications via descriptive analysis and offer suggestions to pharmaceutical companies regarding the selection of drug specifications. The most commonly used specifications for Iohexol Injection are 50 mL and 90 mL, accounting for 42.05% and 56.07% of all Iohexol prescriptions respectively. The most commonly used specification for Iomeprol Injection is 70 mL, accounting for 66.26%. The most commonly used specification for Iopamidol Injection is 80 mL, accounting for 78.95%. The most commonly used specification for Iodixanol Injection is 90 mL, accounting for 87.38%. The most commonly used specification for Ioversol Injection is 80 mL, accounting for 85%. The most commonly used specification for Iopromide Injection is 80 mL, accounting for 78.05%.

## Discussion

In this study, the optimization of the CT contrast-agent workflow and vial-sharing strategy have reduced contrast-agent waste, saved costs, and reduced the economic burden on patients. A total of 2,906,398.8 mL of contrast agent was saved during the one-year study period, corresponding to direct medication cost savings of CNY 9,181,958.83. On average, the strategy saved patients CNY 66.93 per prescription. In 2023, the Chinese government issued the “Implementation measures for saving drug resources and containing drug waste“ [13]. From an institutional standpoint, vial-sharing strategy of contrast agents reduces waste, improves drug utilization efficiency, and saves medical resources. It is also a direct measure aligned with these national healthcare directives. Concurrently, from the patient perspective, vial-sharing strategy reduces patients’ expenses, while CT process optimization transformed manual scheduling into online scheduling. Vial-sharing strategies are widely used in the practice of rational distribution of drugs to minimize waste and lower costs. For example, implementing vial-sharing for bevacizumab resulted in substantial savings in public healthcare expenditures [15]. Baan et al. [26] found that vial-sharing of high-cost drugs such as rituximab, pemetrexed, bevacizumab, and trastuzumab can help reduce drug wastage, offering both economic and environmental benefits. The combination of real-time vial sharing and a daily-rate charge mode decreases both drug wastage and patient expenses [18]. These cases demonstrated that the economic and practical advantages of vial-sharing strategy, while also highlighting their potential environmental benefits. Also, concentrating medication on the same day is also one of the sharing methods. Jiang et al. [27] implemented a cost-saving approach by scheduling multiple patients receiving cabazitaxel treatment on the same weekday to optimize drug use and minimize wastage.

The top 3 largest saved volumes and top 3 cost-saved proportion drugs were Iomeprol Injection (33.36%, 48.97%), Iopromide Injection (27.36%, 28.66%), and Iodixanol Injection (III) (10.40%, 6.09%). The wide variation in cost-saving proportions was primarily attributable to mismatches between commercially available vial specifications and actual clinical patient requirements. For instance, the high cost-saving ratio observed in Iomeprol Injection (48.97%) reveals that its standard market packaging exceeded the average single-patient volumetric demand, leading to extensive residual wastage under the traditional single-vial billing mode. In this case, besides vial-sharing, dose rounding, dose banding and fixed-dosing strategies are measures related to drug dosage in the rational distribution practice of drugs. Dose rounding at the Moroccan Oncology Institute saved USD 75,598.5 over six months by reducing the waste of cytotoxic drugs [17]. Dose banding entailed no significant increase in interindividual plasma exposure, reduced patient waiting time, improved pharmacy planning capacity, and reduced drug wastage [28]. Fixed-dosing has been demonstrated to be valuable, particularly for monoclonal antibodies that target the immune system [29]. Shortliffe et al. [12] called on the FDA and manufacturers to encourage the use of fixed dose or other methods to minimize discarded drugs. These findings may also encourage manufacturers to develop vial sizes that better match clinical requirements.

This study also analyzed contrast agent specifications within a vial-sharing model to provide data-driven sizing recommendations for pharmaceutical manufacturers. For example, it revealed that 90 mL vials may be the most appropriate specification for Iodixanol Injection. Furthermore, the partial factor causing waste of drugs is the mismatch between the specifications of the drug vials and the used doses in clinical practice, resulting in a large amount of drugs remaining in the vials. To mitigate such waste, we may either formulate more rational packaging specifications or use product presentations with validated longer in-use periods, where permitted by product labeling. Drug vial optimization (DVO) would improve dispensing efficiency in medical institutions and reducing drug costs. Nakamura et al. [30] investigated the most efficient vial sizes for infliximab injection and suggested that a 15 mg vial size would be optimal for minimizing drug waste. This optimisation can substantially alleviate the cost burden on the national healthcare system. For contrast agent, Robinson et al. [22] indicated that using 500 mL multiple-use packaging of contrast material could achieve cost containment while enhancing the quality of contrast enhancement. By selecting optimal drug specifications and more favorable drug formulations, the goal of reducing drug waste and saving costs could be achieved.

As for extending the storage time of unused drugs in opened vials, Closed System Transfer Devices (CSTDs) not only mitigate occupational exposure risks and enhance operator safety, but they also preserve the physical, chemical, and microbiological stability of sterile formulations, thereby extending their post-puncture beyond-use dates. In oncology settings, the implementation of CSTDs has been proven to effectively minimize hazardous drug waste, translating directly into measurable financial savings [31]. However, it could be noted that appropriate products should be selected to achieve the intended purpose when using CSTDs. A study in Malaysia [25] demonstrated that the use of CSTDs significantly reduced drug wastage. However, cost savings were not observed, likely due to the extensive use of lower-priced generic drugs. Furthermore, the application of intelligent dispensing technology has played an important role in reducing drug waste. Liu et al. [32] employed a real-time vial-sharing strategy utilizing an intelligent robot to dispense multiple prescriptions. They found that this approach not only saved medical resources, but also alleviated the burden on patients, medical establishments, and the national medical insurance system.

With the development of medical imaging technology, the use of contrast agents is becoming increasingly widespread. Previously, contrast materials, being classified as drugs, were predominantly available in single-use packages. However, the current focus on cost containment, waste reduction, and workflow efficiency has heightened the demand for multiple-use packages of contrast materials. Recent studies have explored various strategies to optimize the use of contrast agents, aiming to reduce costs and waste while maintaining diagnostic quality. Wei et al. [19] showed that multidose packaging of contrast media for contrast-enhanced CT (CECT) is more cost-effective than single-dose packaging. They also found that implementing a multidose bulk IV contrast delivery system, along with varied reimbursement units, reduced waste and led to estimated savings of $12.84 to $14.66 per examination from the patient’s perspective. Arana et al. [33] conducted a study in Spain, demonstrating that a weight-adjusted dose of 1.75 mL/kg of contrast material not only ensures optimal diagnostic quality but also achieves a cost saving of €1.34 per patient. Davenport et al. [34] conducted a retrospective cohort study in the United States, comparing fixed-volume and weight-based dosing regimens for contrast-enhanced abdominopelvic CT. They discovered that using lesser-volume, higher-concentration contrast material had the most significant impact on cost reduction. By implementing a weight-based dosing strategy and reducing the maximum volume of administered contrast material, substantial cost and material savings can be achieved. Also, Routhier et al. [35] reported significant cost savings by transitioning from 100-mL single-use IV contrast vials to a multidose bulk IV contrast delivery system. The change resulted in an average annual reduction in contrast material usage and associated cost savings of approximately $31,800. Moreover, by adopting 500-mL multiple-use packaging of contrast material, technologists can precisely load the required volume of contrast agent for each examination, achieving cost control while ensuring the quality of the contrast agent [22]. These studies collectively suggest that optimizing the use of contrast agents through multidose packaging, weight-based dosing, and careful protocol design can lead to significant cost savings and reduced waste without compromising diagnostic outcomes. Our study also identified the most frequently used dose volumes to inform recommendations for vial sizes that better match clinical practice.

While the economic viability and waste reduction capabilities of the volume-based vial-sharing model are clear, these benefits cannot be evaluated in isolation from clinical safety. In day-to-day practice, the primary barrier to institutional adoption of vial-sharing is the risk of microbial contamination. Uncontrolled multi-dose utilization of preservative-free contrast media frequently can pose a risk of healthcare-associated infection if strict aseptic boundaries are breached. The cost-savings achieved in this study were strictly predicated upon a strict aseptic handling framework designed to minimize contamination risk. By shifting the dispensing site from an open-air radiology suite to a contrast preparation room, the on-site working environment is a more controlled preparation environment. The rubber stopper was disinfected with 75% alcohol before each puncture. Furthermore, the use of a new sterile fluid-transfer spike can maintain the sterility of the withdrawn contrast medium. Normally, the puncture operation is performed no more than five times to ensure the sealing integrity after puncture [36]. Strict adherence to these protocols and the product-specific permitted in-use period limit enables safe reduction of drug waste. These findings indicated that future implementations must prioritize these microbiological checkpoints as prerequisite conditions before expecting reproducible economic returns.

Furthermore, this study is not without its limitations. This study was conducted at a single center and did not include data from other hospitals. The specific operational characteristics of this hospital may not be representative of other healthcare institutions. Future studies should consider a multi-center approach to validate our findings across diverse settings and populations. Additionally, this study did not perform a formal safety, sterility, or stability evaluation. Microbiological culture, endotoxin testing, particulate testing, physicochemical stability testing, and patient-level safety outcomes were not systematically collected and were therefore not analyzed. Although the vial-sharing workflow was implemented with aseptic handling procedures and a strict permitted in-use period for opened vials, future prospective studies should systematically assess microbiological sterility, physicochemical stability, and patient-level adverse events to further evaluate the safety of contrast-agent vial-sharing. While our study demonstrated cost savings through vial-sharing, the economic analysis was based on the specific pricing and usage patterns observed in our hospital. This study did not perform a full health economic evaluation. The analysis focused only on direct medication expenditure and estimated contrast-agent volume saved. Other cost components associated with implementation and operation of the vial-sharing model, including staff time, transfer devices, syringes, tubes, IT system modifications, training, and monitoring were not included. In addition, the broader economic impact related to the disposal and destruction of unused contrast agents was not quantified. Pharmaceutical waste requires specialized handling and incineration, which incurs additional costs and environmental burdens. Future prospective studies should incorporate these additional cost components to evaluate the net economic impact of contrast-agent vial-sharing more comprehensively. Variations in drug costs, reimbursement policies, and healthcare financing models in other regions or countries may affect the replicability of our cost-saving estimates. Despite these inherent limitations, our study provides valuable empirical insights into the capacity of integrated vial-sharing strategies to concurrently mitigate pharmaceutical waste and alleviate financial burdens for both healthcare institutions and patients.

## Conclusion

By optimizing the CT workflow through multidisciplinary collaboration and changing from single-vial to vial-sharing billing, the hospital saved 2,906,398.8 mL of contrast agent and CNY 9,181,958.83 in direct medication costs during one year, and the mean saving was CNY 66.93 per prescription. The vial-sharing strategy substantially reduced drug waste and patients’ financial burden while improving drug utilization and conserving medical resources.

## Data Availability

The datasets generated during and/or analyzed during the current study are available from the corresponding author on reasonable request.

## Abbreviations

CT: computed tomography
HIS: Hospital Information System
NHS: National Health Service
FDA: U. S. Food and Drug Administration
CSTDs: Closed system drug transfer devices
DVO: Drug vial optimization
CECT: Contrast-enhanced CT
CNY: Chinese yuan
